# Editorial: Psychological Sleep Studies: New Insights to Support and Integrate Clinical Practice Within the Healthcare System

**DOI:** 10.3389/fpsyg.2022.857433

**Published:** 2022-02-16

**Authors:** Chiara Baglioni, Luigi De Gennaro, Dieter Riemann, Dagmara Dimitriou, Christian Franceschini

**Affiliations:** ^1^Department of Human Sciences, University of Rome Guglielmo Marconi, Rome, Italy; ^2^Department of Psychiatry and Psychotherapy, Medical Center – Faculty of Medicine, University of Freiburg, Freiburg im Breisgau, Germany; ^3^Department of Psychology, University of Rome Sapienza, Rome, Italy; ^4^Sleep Education and Research Laboratory, Psychology and Human Development, University College London Institute of Education, London, United Kingdom; ^5^Department of Medicine and Surgery, University of Parma, Parma, Italy

**Keywords:** sleep, sleep disorders, clinical psychology and health, insomnia, dreams, narcolepsy, obstructive sleep apnea

Adequate sleep is essential for good health, physical functioning, cognitive performance, and self-regulatory processes. In contrast, sleep disorders affect multiple aspects of an individual's life, including daytime activity, social interactions, mood, and quality of life, playing a role in the whole healthcare public system. Consistently, sleep disorders, especially insomnia, have been reported to be key risk factors for mental and somatic disorders. The International Classification of Sleep Disorders (ICSD-3rd edition, American Academy of Sleep Medicine, 2014) includes six main clinical divisions: Insomnia, Sleep Related Breathing Disorders, Central Disorders of Hypersomnolence, Circadian Rhythm Sleep-Wake Disorders, Parasomnias, and Sleep Related Movement Disorders. Currently clinical practice is often lacking of systematic consideration of psychological aspects related to sleep disorders. Specifically:

(1) Health primary care does not include standard assessment and consequent treatment of insomnia in both pediatric and adult patients, in the general population or in patients with mental and somatic disorders.
(2) Motivational and psychological aspects of sleep disorders and their treatment are often not assessed and treated adequately.

Potential sleep disorders can interfere with current therapies for a variety of different illnesses, and when treated can improve quality of life and adherence to therapy, as well as severity of psychological symptoms presented by the patient. A complex, likely bidirectional relationship, is documented between sleep and mental health problems, including depression, anxiety, and traumatic stress disorders. Clinically there is an increasing demand for psychologists with expertise in evidence base psychotherapy to join multidisciplinary health teams, providing cognitive-behavioral intervention and support for insomnia, narcolepsy, and adherence to treatment for obstructive sleep apnea.

This Research Topic (RT) brought together heterogeneous cutting-edge research on sleep and its disorders in clinical contexts (e.g., treatments, epidemiology), considering both the general population and clinical patients as well as pediatric and adult samples. Sleep, as a main aspect of physical and mental health, interacts with other processes strictly associated with wellbeing and quality of life. Two studies collected in the present RT deepened the relationship between sleep quality and other indices of health or quality of life, such as physical activity, wellbeing and academic performance (Dubinina et al.; Armand et al.). Both works interestingly pointed out complex interactions between variables, suggesting that multiple factors intervene in defining individual's health patterns. Epidemiological studies are necessary to capture these complex interactions, which may explain individual variability. Specialists should be aware of the different pathways to tailor their intervention to individual's needs, thus, providing more efficacious guide to patients in finding optimal balance for their health and wellbeing. Clinically, sleep should be assessed systematically being fundamental for brain functioning. As Holzinger et al. point out insomnia and nightmares are common experience in patients with psychiatric illness, though often not assessed nor treated. The authors, by reporting an example of a case study of a woman diagnosed with Post-Traumatic Stress Disorder (PTSD), propose a program including psychological treatment for insomnia symptoms and lucid dreams training as clinical module to be offered in psychiatric care.

Studies dealing with sleep and psychological variables during adolescence are of utmost importance right now. Indeed, sleep goes through dramatic changes during adolescence determined both by biological and social developments. A shift for a more delayed circadian preference is often problematic due to school starting times. The pandemic outbreak and its social consequences particularly affected adolescents' mental health and sleep functioning (e.g., Becker and Gregory, 2020). In the present RT, Haugland et al. provided a contribution to this literature showing extremely high rates of insomnia and sleep difficulties and strict association of sleep problems with psychopathological symptoms in an adolescent sample referred to anxiety treatment. Sleep and circadian preference during adolescence should be systematically assessed and treated in primary care. School preventive programs should be encouraged too.

Several specific clinical populations were considered in the RT. Specifically, two studies focused on treatment for sleep problems directed to parents of children suffering of epilepsy, a population which is highly afflicted by insomnia and difficulties sleeping. While standard protocols exist for behavioral interventions for sleep problems during development, often they do not include adaptation for specific clinical populations. Cook et al. and Wiggs et al. provide data of how protocol adaptations for specific clinical populations may be created with the help of patients (in this case parents of children with epilepsy), thus, offering modules which satisfy individual's wishes and may promote better motivation and adherence to prescriptions. Considering adults, two studies contributed to the understanding of the relationship between sleep quality and pain. Zambelli et al. reported on a large sample of 1,234 participants with chronic pain that better sleep quality was associated to a reduced link between depression and pain. Blytt et al. nevertheless, found in patients with dementia that those reporting more pain had actually longer sleep times within the time the spent in bed as registered with actigraphy. Again, complex interactions between variables are suggested by results of empirical studies. While sleep patterns should be more carefully assessed and considered clinically, research should be strengthened to deepen different individual pathways.

Psychological interest in sleep research is often directed to the disorder of insomnia and to the link between sleep and mental health. Nevertheless, another area of great interest is how psychological variables associate to sleep disorders and how motivational and psychological interventions can contribute to medical treatment of sleep disorders. As two studies in the RT point out Narcolepsy type 1 (NT1), a neurological chronic disease associated with altered sleep structure and behavior, is characterized by cognitive and emotional dysfunctions. The works by Filardi et al. and Scarpelli et al. deepened sleep, psychological, and psychopathological variables in this disorder through, respectively, the example of a case of an adolescent girl and a study comparing patients and controls during the pandemic period. Psychological interest in sleep research is growing and expanding to all areas of interest. This should correspond to a more standardized inclusion of clinical psychologists/psychotherapists in sleep medicine teams, as well as a regular interest in sleep variables in psychotherapy.

Continuous positive airway pressure (CPAP) therapy is the standard treatment for obstructive sleep apnea (OSA) syndrome. However, optimizing adherence to CPAP therapy of individuals remains very challenging for clinicians because of the role played by the psychological components. Two studies considered this issue. Scarpina et al. found that a psychological training assessing risks and benefits of CPAP therapy improved adherence to treatment. Consistently a review of Rapelli et al. about motivational intervention for CPAP therapy support their integration in care for OSA patients. A final paper in the RT by Galbiati et al. deepened the mechanisms underlying psychological treatment for insomnia. Cognitive Behavior Treatment for Insomnia (CBT-I) is recognized as first line intervention for patients with insomnia comorbid and non-comorbid with other disorders (e.g., Riemann et al., 2017). Though, there is a need to explore more in details what are the mechanisms explaining its efficacy. The authors reported results from group therapy showing that the intervention reduced nighttime symptoms of insomnia and dysfunctional beliefs about sleep in patients. However, patients did not change the way they reacted on their own thoughts about sleep loss and its consequences. These secondary cognitions, often exacerbating the disorder, are indeed not systematically considered in standard CBT-I protocols. Emergent protocols, as the Mindfulness Based Treatment for Insomnia (MBTI) proposed by Ong et al. (2012), direct their attention to aspects associated with secondary arousal and cognitions, and it has been suggested to improve CBT-I efficacy by including more attention to daytime symptoms and emotional variables. Nevertheless, empirical data are not yet stable enough (e.g., Baglioni et al., 2020).

Though heterogeneous, and covering several different topics, all papers included in the RT support the relevance of sleep assessment and treatment in primary and secondary care. Future research should deepen further the complex interaction between health variables in specific clinical population to guide protocols which can be tailored to individual's needs. All together the papers of this RT give support and suggestion to improve clinical and healthcare attention to sleep.

## Author Contributions

All authors listed have made a substantial, direct, and intellectual contribution to the work and approved it for publication.

## Conflict of Interest

The authors declare that the research was conducted in the absence of any commercial or financial relationships that could be construed as a potential conflict of interest.

## Publisher's Note

All claims expressed in this article are solely those of the authors and do not necessarily represent those of their affiliated organizations, or those of the publisher, the editors and the reviewers. Any product that may be evaluated in this article, or claim that may be made by its manufacturer, is not guaranteed or endorsed by the publisher.
